# Detecting Soldiers’ Fatigue Using Eye-Tracking Glasses: Practical Field Applications and Research Opportunities

**DOI:** 10.1093/milmed/usab509

**Published:** 2021-12-14

**Authors:** Theresa Schweizer, Thomas Wyss, Rahel Gilgen-Ammann

**Affiliations:** Monitoring, Swiss Federal Institute of Sport Magglingen (SFISM), Macolin 2532, Switzerland; Monitoring, Swiss Federal Institute of Sport Magglingen (SFISM), Macolin 2532, Switzerland; Monitoring, Swiss Federal Institute of Sport Magglingen (SFISM), Macolin 2532, Switzerland

## Abstract

**Introduction:**

Objectively determining soldiers’ fatigue levels could help prevent injuries or accidents resulting from inattention or decreased alertness. Eye-tracking technologies, such as optical eye tracking (OET) and electrooculography (EOG), are often used to monitor fatigue. Eyeblinks—especially blink frequency and blink duration—are known as easily observable and valid biomarkers of fatigue. Currently, various eye trackers (i.e., eye-tracking glasses) are available on the market using either OET or EOG technologies. These wearable eye trackers offer several advantages, including unobtrusive functionality, practicality, and low costs. However, several challenges and limitations must be considered when implementing these technologies in the field to monitor fatigue levels. This review investigates the feasibility of eye tracking in the field focusing on the practical applications in military operational environments.

**Materials and Method:**

This paper summarizes the existing literature about eyeblink dynamics and available wearable eye-tracking technologies, exposing challenges and limitations, as well as discussing practical recommendations on how to improve the feasibility of eye tracking in the field.

**Results:**

So far, no eye-tracking glasses can be recommended for use in a demanding work environment. First, eyeblink dynamics are influenced by multiple factors; therefore, environments, situations, and individual behavior must be taken into account. Second, the glasses’ placement, sunlight, facial or body movements, vibrations, and sweat can drastically decrease measurement accuracy. The placement of the eye cameras for the OET and the placement of the electrodes for the EOG must be chosen consciously, the sampling rate must be minimal 200 Hz, and software and hardware must be robust to resist any factors influencing eye tracking.

**Conclusion:**

Monitoring physiological and psychological readiness of soldiers, as well as other civil professionals that face higher risks when their attention is impaired or reduced, is necessary. However, improvements to eye-tracking devices’ hardware, calibration method, sampling rate, and algorithm are needed in order to accurately monitor fatigue levels in the field.

## INTRODUCTION

Fatigue inhibits optimal cognitive and physiological functioning.^1,2^ In military environments, fatigue is very common due to high mental and physical strain, high pressure, and a low quantity and low quality of sleep but can also appear more individually due to high stress, depression, or a recent head impact.^3–6^ Reduced cognitive and physical capacities may decrease individuals’ ability to process information and respond adequately to hazardous situations. Accordingly, fatigue is known to contribute to human errors, injuries, accidents, and other health issues.^7,8^

Quantifying fatigue in operational settings can provide actionable and vital information about the individual to improve safety and effectiveness during training or operations. Rather than extrapolating from external conditions (e.g. weather, activity) and assuming typical responses of the person to these conditions, tracking individual’s actual readiness provides an internal picture of each soldier and enables adequate action or choose the right countermeasure to reduce fatigue and enhance performance.^9^

Fatigue is often measured subjectively through self-reported ratings.^10,11^ Although subjective measures provide insights into an individual’s fatigue impressions and perceptions, biases are associated with the use of self-reports, potentially leading to erroneous results (e.g., moods, the desire to please, interests, or opinions).^11^

Notably, the regulatory process of fatigue can be unconscious, and cognitive and physical performance can decrease before a fatigued individual is aware of being tired.^12^ Moreover, intrinsic or extrinsic motivations or pressures can push such individuals to exert themselves beyond their bodies’ capacities, concealing the sensitivity of their fatigue.^13^ In such cases, subjective fatigue ratings may underestimate performance deficits, limiting the efficacy of fatigue self-reports or one’s own decision to pause when fatigued. Therefore, the objective monitoring of physiological biomarkers to assess individuals’ fatigue levels could present an effective approach to preventing overload and—more importantly—its consequences. For instance, various biomarkers have been studied, including cardiac dynamics using an electrocardiogram,^14,15^ electrical brain activity using an electroencephalogram,^16,17^ brain hemodynamic responses using functional near infrared spectroscopy,^18^ and oculometrics using eye tracking.^19–23^ These biomarkers could monitor fatigue, but accurate assessments of their use in this context have been lacking in the literature, especially in real-life settings.^24^

Only a few existing methods can provide objective, field-ready, unobtrusive, and continuous assessments of individuals’ fatigue levels. In this regard, eye-tracking glasses are practical, low-cost, unobtrusive, and able to provide objective information about an individual’s cognitive state.^25–27^ Therefore, eye-tracking glasses can be practically applied to military settings for soldiers’ use as protective glasses during their duties. However, Schweizer et al.^28^ demonstrated that eye-tracking glasses do not validly or reliably track eye movements in field settings during military tasks. In contrast, when investigated in labor contexts, eye trackers have been found to achieve good reliability in eye-movement tracking.^19,26,29^ Evidently, the performance gap between tracking eye movements in controlled or unnatural settings and tracking such movements in unconstrained operational environments is considerable. To implement these systems in real-life settings in order to monitor fatigue levels, several challenges must be considered. Therefore, the current paper addresses the challenges and limitations of eye-tracking systems and eyeblink detection under field conditions. First, different oculometric biomarkers are described, feasibility in the field is evaluated, and research gaps in literature are discussed. Second, optical eye tracking (OET) and electrooculography (EOG) technologies are described, and an overview of the currently available wearable eye-tracking glasses is provided. Third, eye-tracking systems’ feasibility in the field is discussed, and recommendations to improve feasibility are given, such as a recommended sampling rate and various software and hardware features.

## OCULOMETRICS AS BIOMARKERS

This section describes various oculometrics attributed to eyeblink and fatigue and outlines particular concerns and gaps in the literature. First, eye tracking provides information about oculometrics (i.e., quantitative indices characterizing eye movements^25^) that may be perceived as reflecting the underlying neural mechanism.^30^ Oculometrics have been studied for decades, and their relation to fatigue has been widely confirmed.^20,24^ Additionally, the development of fatigue has been shown to possibly manifest earlier in oculometrics than in physical and cognitive performance during different tasks.^24^ Therefore, oculometrics can be regarded as promising biomarkers, and they are accepted as sensitive metrics for the early detection of fatigue.

Among the various eye movements (e.g., saccades, fixations, and eyeblinks), spontaneous or endogenous eyeblink is the dominant ocular event in the literature. It offers various advantages, such as ease of observation, a lack of necessary external stimuli, and known correlations with fatigue development.^29^ Eyeblink can be seen as rapid eyelid closing and opening movements, mainly to clean and lubricate the surface of the cornea and conjunctiva. This blink activity, and especially endogenous blinking, has been repeatedly found to reflect cognitive states; therefore, it is widely used as an indicator in fatigue diagnostics.^31^  Table I presents oculometrics that studies have attributed to eyeblinks.

**TABLE I.** The Main Oculometrics Attributed to Eyeblinks, Measured Using Eye-tracking Technology

Eyeblink metric	Abbreviation (unit)	Operational definition	Changes with fatigue
Blink frequency or rate	BF or BR (blinks/min)	Number of blink occurrences per minute	Increases
Blink duration	BD (s)	Total time from the start to the end of a blink	Increases
Blink interval	BI (s)	The time between 2 blinks	Decreases

Blink frequency (BF) is the most accepted indicator of fatigue development.^23,24,29,32^ Zargari Marandi et al.^29^ explained the increase in BF as the result of deactivated blink inhibition due to decreased cognitive and attentional resources as fatigue develops. Thus, BF is associated with the onset and development of fatigue. Yet, the number of blinks per minute can be reasonably presumed to achieve a maximum value while fatigue might continue to increase.^22^ Furthermore, some concerns can complicate the study of BF, such as situational variations in BF. Studies have shown that BF generally increases as more time is spent on a task, while the mind wanders, and during conversations. By contrast, during more focused periods and highly visually demanding test segments (e.g., reading or particularly demanding flying tasks), BF tends to decrease.^24,33–36^ Further, individuals with dry eyes have been shown to blink more frequently than individuals without dry eyes, whereas no BF differences were observed between young and elderly subjects.^29,37^ Additionally, BF depends on environmental conditions, such as temperature, relative humidity, wind, flying debris, and lighting conditions.^20^ Thus, although BF is a very good fatigue indicator, various complicating factors must be considered—especially when assessing BF in real-time field environments.

Blink duration (BD) is considered one of the most robust sleep-related metrics.^21,29,35,38–40^ A few studies have divided BD into 3 phases—blink closure duration, blink reopening duration, and the duration of the period between closure and reopening (i.e., the lid reopening delay)—and these phases were described as sensitive indicators of changes to an individual’s readiness.^19,41,42^ Indeed, when an individual is fatigued, the closing and reopening of their eyelids slows, and their eyelids remain closed longer, increasing total BD. This increase might be caused by the inhibited tonic contraction of the levator palpebrae muscles, which reduces this muscle’s strength and slows blinking.^43^ Yet, to the author’s knowledge, these 3 separate metrics have never been investigated in field measurements using head-mounted eye trackers. Presumably, such an investigation is lacking because it requires a very high sampling frequency and highly precise measurements. Furthermore, some recent studies have noted strong variability in BD among subjects.^22,44^ Ftouni et al.^44^ measured BD over 40 hours of wakefulness, observing significant variability in BD as sleepiness increased. Although BD did not significantly increase at the end of that study’s assessment period, the authors noted that BD still classified attention lapses with good accuracy. Thus, BD is accepted as a good indicator of fatigue development; however, significant intra- and inter-variability can occur during long BD measurements.

Blink intervals (BI) have also been analyzed in a few studies during the early 21st century. Although BI appeared to decrease as fatigue increased (an obvious correlation since BF increases with fatigue), this decrease was insignificant and susceptible to outliers, as well as strong individual variations.^19,45^ Further research in this context is, therefore, needed.

Overall, BF and BD are the 2 most commonly used oculometrics that are simple to obtain using eye-tracking systems and that represent good indicators of increasing fatigue. However, to determine fatigue levels using BF or BD, several factors must be accounted for. For instance, eyeblink dynamics are affected by lighting conditions, wind, flying debris, temperature, humidity, sweating, and an individual’s general situation. Therefore, a subject’s environment, situation, and behavior must be included in the equation when assessing eye blinks in the field. To the author’s knowledge, a valid threshold of BF or BD that corresponds to fatigue has not yet been reported for field measurements, and such a threshold appears very challenging to determine.

## EYE-TRACKING SYSTEMS

The common wearable eye-tracking systems use OET technology or EOG. The current section describes these 2 systems and enumerates particular considerations related to these technologies.

The OET device uses miniature eye cameras mounted on frames that are placed either at the inner canthus of the eye, adjacent to the bridge of the nose, or at the outer canthus of the eye. This device captures images of the eyes either monocularly or binocularly, and directly or indirectly; if indirectly, the device applies infrared-reflective mirrors in front of the eyes.^46^ To detect a wearer’s gaze in their current field of view, an additional camera (or scene camera) may be mounted at the outside of the frame to film in front of the wearer. The OET can be categorized into *passive illumination* and *active illumination* systems. Active illumination systems use a combination of infrared light-emitting diodes to illuminate the eye as well as daylight filters to block any other-than-infrared radiation from the camera lens. This method assures high-contrast images in all lighting conditions.^46^ Meanwhile, the less common passive illumination systems use visible light, which can distract the wearer and display lower contrast of the pupil than active illumination devices.^47^ Currently, to process and save raw recorded images, eye-tracking glasses are connected to a compact computer that is worn on the device-wearer’s hip or in a backpack. A key factor in eye-tracking systems’ reliability and validity is their recordings’ sampling rate. Kredel et al.^46^ noted that, until 2017, the median sampling rate for mobile OET devices was 30 Hz while the maximum stood at 60 Hz. Today, the OET technology available on the market reaches up to 200 Hz; however, current rates range between 50 and 200 Hz (Table II).

**TABLE II.** Overview of the Currently Available Wearable Eye-tracking Glasses

Image	Model (year of release)	Company (country)	Eye-tracking technology (OET/EOG)	Calibration procedure	Slippage compensation	Lens material	Sampling rate (Hz)	Battery recording time
	Tobii Pro Glasses 3 (2020)^57^	Tobii Pro AB (Sweden)	OET (binocular, 4 infrared eye cameras)	One-point	Yes, 3D eye-tracking model	Optical-grade plastic	50 Hz/100 Hz	105 min
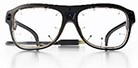								
	Pupil Labs Core (2014)^58^	Pupil Labs GmbH (Germany)	OET (binocular, 2 infrared eye cameras)	Five-point/multiple-point	Yes, 3D eye-tracking model	No lenses	200 Hz	N/A
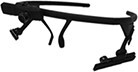								
	Pupil Labs Invisible (2019)^59^	Pupil Labs GmbH (Germany)	OET (binocular, 2 infrared eye cameras)	None	Slippage invariant	CR-39	66 Hz/200 Hz	150 min
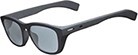								
	VPS19 (2019)^60^	Viewpointsystem GmbH (Austria)	OET (binocular, 2 infrared eye cameras)	Yes	N/A	N/A	60 Hz	>480 min (with battery swap)
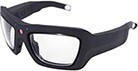								
	ETVision (2019)^61^	Argus Science LLC (United States)	OET (binocular, 2 infrared eye cameras)	One-point/multiple-point	N/A	N/A	180 Hz	300 min
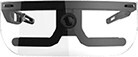								
	AttentivU new version (2019)^62^	Massachusetts Institute of Technology Media Lab Fluid (United States)	EOG (2 electrodes as nose pads and one placed on the bridge of the nose)	N/A	N/A	N/A	1000 Hz	450 min
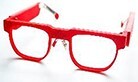								
	J!NS MEME (2014)^63^	J!NS MEME (Japan)	EOG (2 dry electrodes as nose pads and one placed on the bridge of the nose)	None	N/A	Plastic	100 Hz/200 Hz	960 min
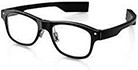								

Abbreviations: N/A = not available (information could not be found); OET = optical eye tracking; EOG = electrooculography.

The traditional method for recording EOG signals places electrodes around the wearer’s eye or on their forehead; thus, the majority of studies have not used EOG glasses to monitor eye movements.^18,48–51^ However, EOG glasses are becoming more common, and a few prototypes have been developed. Despite this growing interest, to the best of the author’s knowledge, only a few studies have used EOG glasses to monitor eye movements.^23,52,53^ EOG glasses generally include 3 dry electrodes that are placed around the wearer’s nose.^23,54^ Since the eyeball constitutes an electrical dipole with a positive pole at the cornea and a negative pole at the retina, EOG measures changes to corneoretinal potential when the eyeball moves. During a blink, the eyes perform a characteristic, downward, nose-oriented motion that can be measured.^23^ EOG technology is, therefore, not affected by sunlight, but the placement of EOG electrodes is crucial, and electrodes must make contact with the wearer’s skin at all times.^55^ EOG has a sampling rate of up to 1,000 Hz (Table II).^49,52,56^

## EYE-TRACKING SYSTEMS’ *FEASIBILITY* IN THE FIELD

Accuracy, validity, reliability, robustness, and suitability are central factors for eye-tracking systems’ application to field measurements. The current section discusses the current state of the art for this usage, as well as its advantages, disadvantages, challenges, and limitations.

The unobtrusiveness, practicability, and low costs of OET and EOG eye trackers are their main advantages for real-world implementation. These glasses can be implemented particularly effectively as protection goggles in military settings because soldiers usually wear goggles already as protection of the eye is essential.

OET works with mounted eye cameras on glasses’ frames, and these cameras’ placement plays a crucial role in such devices’ practicability and feasibility in the field. OET systems with cameras mounted at the inner canthus of the eye, directly embedded into the frame, have proven more robust against impacts, sunlight, and vision losses compared to models with cameras at the outer canthus of the eye, which are usually not embedded into the frame.^58^ Recently, a field study demonstrated that OET struggles, especially with sunlight.^28^ Active illumination systems and high-contrast images of the eye appear to depend on lighting conditions^28^ despite promotions suggesting that they do not. Placing eye cameras at the inner canthus of the eye, embedded into the frame, could enable better shielding from the sun while also creating more robust hardware. Moreover, such wearable devices should come with photochromic lenses that darken with exposure to sunlight, protecting wearers’ eyes from possible reflections.^64,65^

Nevertheless, OET has also seemed to struggle with pupil detection—even without sunlight complications.^28^ Indeed, eye cameras’ sampling rates in most OET systems on the market have been shown to be too low to detect eye movements in real-time and real-life settings.^66^ Recently, Alsaeedi and Wloka^67^ calculated that a minimum of 73 Hz is needed to capture eyeblinks reliably; however, 200 Hz is preferable. Therefore, the use of at least 200 Hz is recommended to detect oculometrics accurately—especially in field tests, in which head movements and walking or running vibrations can blur images of the eyes.^23,28,68^ Additionally, to avoid deteriorating video feed quality (i.e., images of the eyes) due to movements, software must be updated. For example, in 2020, a previously tested eye-tracking algorithm was introduced that is known to offer robust performance against slippage.^69^ Indeed, the pupil detection algorithm is a core component of all eye trackers that must operate robustly and accurately in all conditions. However, pupil detection with head-mounted eye trackers has been challenging due to fundamental limitations that must be resolved before practical applications under field conditions.^28,46,64,65^

EOG glasses work with mounted electrodes on their frames. These electrodes are gel-free and, therefore, represent an advantage compared to conventional EOG signal acquisition systems, which require gel for conductivity.^26^ Tag et al.^23^ used EOG glasses to identify fatigue in real-life daily activities over 14 days. Their report showed promising results regarding the unobtrusive and continuous monitoring of wakefulness throughout the day. However, the electrodes connected to the studied frames were found to depend heavily on the glasses’ proper placement on a wearer’s nose. When wearers touched their faces, moved quickly, turned their heads rapidly, or even used their facial muscles intensely, the EOG signals became noisy.^23^ Deng et al.^68^ reported that a subject’s nervousness, exaggerated gestures, speech, and sweaty skin affected eye-movement detection. Therefore, EOG’s implementation in daily life measurements remains challenging. Nonetheless, EOG offers 2 advantages over OET. First, it is robust against glaring sunlight since it uses no images of the eyes to detect pupils and calculate eyeblinks. Second, it usually has a higher sampling rate than OET.

For both OET and EOG technologies, calibration is among the most important factors in achieving accurate eye tracking, adjusting for the individual differences caused by either geometric characteristics of the subject’s eyes or glasses’ placement.^70,71^

Indeed, the devices’ movement or displacement during the activities or exercises can lead to calibration drifts—a cumulative deterioration in eye-tracking accuracy during continuous monitoring.^72^ Currently, some devices are calibrated manually and only once, at the beginning of a measurement. Automatic calibration might enhance eye-tracking systems’ performance through frequent automatic adjustments during recordings or measurements. Interestingly, automatic calibration (i.e., without an initial calibration) or recalibration (i.e., with an initial calibration) could overcome the challenges of such devices’ movements, thereby enhancing eye-tracking accuracy.^70,72^

Moreover, eye-tracking glasses’ implementation in unconstrained operational environments entails a few requirements. Their hardware’s robustness must be considered when targeting field applications. It must be resistant to humidity, rain, wind, extreme temperatures, and impacts. Additionally, eye tracking in real-life settings must be conducted without disturbing wearers’ sight, inducing mental stress, or influencing eyeblink metrics.^23^ Wearers’ safety must also be assured, especially since eye trackers are worn near the eyes, which are fragile. These wearable devices’ design is, therefore, important. Their cameras or electrodes must be part of their frames to avoid piercing the eye in the case of an impact. Also, their lenses must be made of polycarbonate or similar materials to be shatterproof (more resistant than plastic) and photochromic (so that they darken in sunlight and lighten indoors).^73^ The transition between light and dark tints must be rapid, and it could even be controlled by a button at the side of the frame for quick changes if needed. The glasses should also be fog-free and not act as a moisture chamber, avoiding increased humidity under the glasses that could affect eye dynamics.^74^ To ensure that aspects of the device do not hamper its performance, the device must be comfortable and lightweight, without reducing a wearer’s field of view. Finally, since soldiers are outside most of the time—and active in different settings and over long periods—these eyeglasses should be wirelessly connected to recording units (small computers) to avoid restricting wearers’ movements, and their batteries should last at least 24 hours and ideally for several days.^75^ If these wearable devices are thus built intelligently and consciously, they could serve as protective eyewear for soldiers or other operational personal. Moreover, since wearable OET and EOG technologies face different limitations, a combination of both measurement devices might offer a solution, improving accuracy under outdoor conditions. To the author’s knowledge, such a combination of both technologies remains unexplored in the literature and is not available on the market.

These devices could be implemented in more stable environments, such as among medical personnel, drivers, air traffic controls, or pilots, whose gaze remains more stable and whose heads move less than professionals in the field and unconstrained environments.^23,76^ Peißl et al.^76^ noted the potential for eye-tracking devices’ prevention of errors or injuries through detecting fatigue or performance decrements if applied appropriately in simulated or real flights.

## CONCLUSION

The objective physiological monitoring of fatigue levels is necessary for soldiers, as well as other civil professions that face higher risks when their attention is impaired or reduced. This paper has summarized the existing research on eye-tracking technologies under field conditions and included some recommendations. Table III reiterates this paper’s practical recommendations for eye tracking in unconstrained field measurements.

**TABLE III.** Summary of This Paper’s Practical Recommendations and Some Requirements for Eye Tracking under Unconstrained Field Conditions

	OET	EOG
Eye cameras (OET) and electrodes (EOG)	Placement: the inner canthus of the eye Embedded in the frames	Skin-electrode contact Sweat-resistant
Sampling rate	200 Hz	1,000 Hz
Software	Slippage-resistant (algorithms) Movement- and vibration-resistant Automatic calibration Wireless data transfer	
Hardware	Lenses: polycarbonate and photochromic Safe for users Resistant to all outdoor conditions Fog-free and does not act as a moisture chamber Battery recording time: 24 hours	

Abbreviations: OET = optical eye-tracking; EOG = electrooculography.
